# Conservative therapy is associated with worse clinical features and biochemical derangements than renal replacement therapy: a retrospective study in Kumasi, Ghana

**DOI:** 10.1186/s12882-022-02951-z

**Published:** 2022-10-26

**Authors:** Perditer Okyere, Isaac Okyere, Grace Essuman, Joseph Attakora, Dorcas Serwaa, Irene Esi Donkoh, Richard K.D. Ephraim

**Affiliations:** 1 Department of Medicine, School of Medicine and Dentistry, College of Health Sciences, Kwame Nkrumah University of Science and Technology, Kumasi, Ghana; 2 Department of Surgery, School of Medicine and Dentistry, College of Health Sciences, Kwame Nkrumah University of Science and Technology, Kumasi, Ghana; 3 Department of Medical Laboratory Science, School of Allied Health Sciences, University of Cape Coast, Cape Coast, Ghana; 4 Department of Obstetrics and Gynecology, College of Medicine, Institute of Life and Earth Sciences, Pan African University, University of Ibadan, Ibadan, Nigeria; 5 Kidney Research Initiative, Cape Coast, Ghana

**Keywords:** Conservative therapy, Renal replacement therapy, Chronic kidney disease, End stage kidney disease.

## Abstract

**Background:**

The incidence of end stage kidney disease (ESKD) is increasing in Ghana as with the rest of the world. This study compared the sociodemographic, diagnostic characteristics (clinical, biochemical and imaging) and clinical outcomes of ESKD patients who chose either renal replacement therapy (RRT) or conservative therapy as well as the factors that influenced their choice.

**Methods:**

We retrospectively reviewed the records of 382 ESKD patient from 2006 to 2018. The data was collected from the Nephrology Clinic at the Komfo Anokye Teaching Hospital (KATH). Sociodemographic, diagnostic (clinical, biochemical and imaging) and therapeutic data were obtained, organized and analyzed with Statistical Package for the Social Sciences (SPSS).

**Results:**

Of the 382 patients, 321 had conservative therapy whiles 61 had renal replacement therapy. The mean age of participants was 47.71 ± 16.10 years. Bipedal swelling (16.8%), fatigue (10.4%) and facial swelling (9.2%) were the major clinical features. Chronic glomerulonephritis (31.4%), hypertension (30.3%) and diabetes mellitus nephropathy (28.2%) were the most frequent predisposing conditions. Nifedipine (82.0%), bisoprolol (32.8%), aspirin (19.7%), ranitidine (26.2%), metformin (13.1%) and lasix (78.7%) were commonly used by the RRT patients than their conservative therapy counterparts. Compared to their RRT counterparts, patients on conservative therapy were more on irbesartan/lisinopril (57.9%) and sodium hydro carbonate (NaHCO_3_) (52.0%). Diastolic blood pressure (DBP) (p = 0.047), uremic gastritis (p = 0.007), anaemia, uraemia, haematuria and hyperkalaemia (p < 0.001) were more common in conservative therapy patients than RRT patients with RRT patients showing better corticomedullary differentiation (38.1% vs. 27.7%, p < 0.001) and normal echotexture (15.0% vs. 11.6%, p = 0.005). Age, gender, occupation and duration of illness were significantly associated with the decision to opt for conservative therapy.

**Conclusion:**

Patients on conservative therapy have worse clinical outcomes than their RRT counterparts. Early referrals to nephrologist as well as subsidized RRT should be targeted.

## Introduction

The incidence of Chronic Kidney Disease (CKD) is increasing in Ghana [1, 2] as with the rest of the world [3–6]. It is a growing global health issue and is associated with high morbidity and mortality [7]. Risk factors of CKD includes obesity, hypertension, diabetes mellitus, HIV (Human Immunodeficiency Virus) among others [3, 8, 9]. Complications such as gout, anemia, cardiovascular disease occur as the disease progresses which greatly increases morbidity [10, 11].

As CKD advance to end stage kidney disease (ESKD), patients may choose either conservative therapy or renal replacement therapy (RRT) for the continual management. Conservative therapy involves treating symptoms, efforts to control complications and palliative care. This decision, in consensus with caregivers, is ideally chosen for aged patients [12, 13]. RRT includes hemodiafiltration, hemodialysis, kidney transplantation and peritoneal dialysis. These are engaged in critical acute kidney injury, ESKD or in acute kidney injury superimposed on chronic kidney disease. [14]

The management of this condition is highly burdensome as it takes a heavy toll on both the financial and emotional well-being of both patients and loved ones. In sub-Saharan Africa, the outcome of ESKD is rather poor because the resources and expertise needed for combating the disease are scarce and there is limited awareness of the disease and its risk factors. Per se, there is the need to concentrate more efforts on improving the disease outcomes. In some countries, RRT and conservative care regimes are being closely monitored [15–19] to determine which treatment regimen is better suited for their citizens. Currently, no study has been conducted in Ghana to analyze the patterns of ESKD management of which this study sought to accomplish. We compared the sociodemographic features, diagnostic characteristics (clinical, biochemical and imaging) and clinical outcomes of ESKD patients who chose either RRT or conservative therapy as well as the factors that influenced their choice.

## Methodology

### Study design

We retrospectively reviewed the records of 382 ESKD patient from 2006 to 2018. The data was collected from the Nephrology Clinic at the Komfo Anokye Teaching Hospital (KATH). KATH, the second largest public hospital in Ghana with 1000 bed capacity and has one of the only six public dialysis centers in the country.

## Eligibility criteria

ESKD patient records within the period under review was used. CKD patients who had not reached stage 5 ESKD and patients with incomplete data were excluded.

## Data collection

Data of 382 ESKD patients from 2006 to 2018 (male/female) was manually collected from the records of the nephrology unit and sifted through to obtain demographic information including: gender, age, religion and occupation as well as clinical information including: cause of ESKD, symptoms presented, presence or absence of hypertension and/or diabetes mellitus, results of biochemical tests, treatment administered and ultrasound results of each patient.

## Ethical consideration

Ethical clearance for the study was obtained from the research and development unit of KATH. Data used was obtained in adherence to the principles of the declaration of Helsinki and local regulatory requirements. Informed consent was obtained from all participants of the study.

### Data analysis

Statistical Package for the Social Science (SPSS; IBM, USA) Version 22 was used for all statistical analyses. For demographic factors, descriptive statistics was used. For categorical variables, the results were represented as a percentage (%) or a number (n), and for continuous variables, means SD) or median (interquartile range). For categorical variables, the chi-square test or Fisher exact test was used, and for continuous variables, the t test or Mann–Whitney U test was used, as applicable. To find associated factors with outcome variables, a binary logistic regression analysis was performed and all independent variables with p < 0.05 were submitted to a multivariable logistic regression analysis. The adjusted odds ratio was considered statistically significant at p < 0.05 and 95% confidence level in the final model. The threshold for statistical significance was fixed at p < 0.05.

## Results

This study involved 382 ESKD patients of which 61/382(16%) underwent renal replacement therapy (RRT) while 321/382 (84%) were put on conservative treatment. The mean (SD) age of the participants was 47.71 ± 16.10 years. Both RRT and conservative groups had more males (68.9% vs. 57.9%, p < 0.001), Christians (88.5% vs. 91.6%, p < 0.001) and employed within the informal sector (46.4% vs. 57.5%, p < 0.001).

**Table 1:** Sociodemographic characteristics of the study participants

Baseline characteristics	All patients	Renal replacement therapy group	Conservative group	P-value
**Age (years)**	N (%)	N (%)	N (%)	
10–24	45 (11.8)	2 (3.3)	43 (13.4)	< 0.001
25–39	76 (19.9)	12 (19.9)	64 (19.9)	< 0.001
40–54	114 (29.8)	25 (41.0)	89 (27.7)	< 0.001
55–69	118 (30.9)	19(31.1)	99(30.8)	< 0.001
≥ 70	29 (7.6)	3(4.9)	26(8.1)	< 0.001
**Gender**				
Female	154(40.3)	19(31.1)	135(42.1)	< 0.001
Male	228(59.7)	42(68.9)	186(57.9)	< 0.001
**Religion**				
Muslim	34(8.9)	7(11.5)	27(8.4)	0.001
Christian	348(91.1)	53(88.5)	294(91.6)	< 0.001
**Occupation**				
unemployed	62(18.1)	10(17.9)	52(18.1)	< 0.001
students	32(9.9)	3(5.4)	31(10.8)	< 0.001
Pensioners	17(5.0)	2(3.6)	15(5.2)	0.002
Informal sector	192(56.0)	26(46.4)	166(57.8)	< 0.001
Formal sector	38(11.1)	15(26.8)	23(8.0)	0.194
**Duration of illness (months)**				
< 1	46(24.3)	10(47.6)	36(21.4)	< 0.001
1–5	119(63.0)	8(38.1)	111(66.1)	< 0.001
> 6	24(12.7)	3(14.3)	21(12.5)	< 0.001

**Fig. 1:**
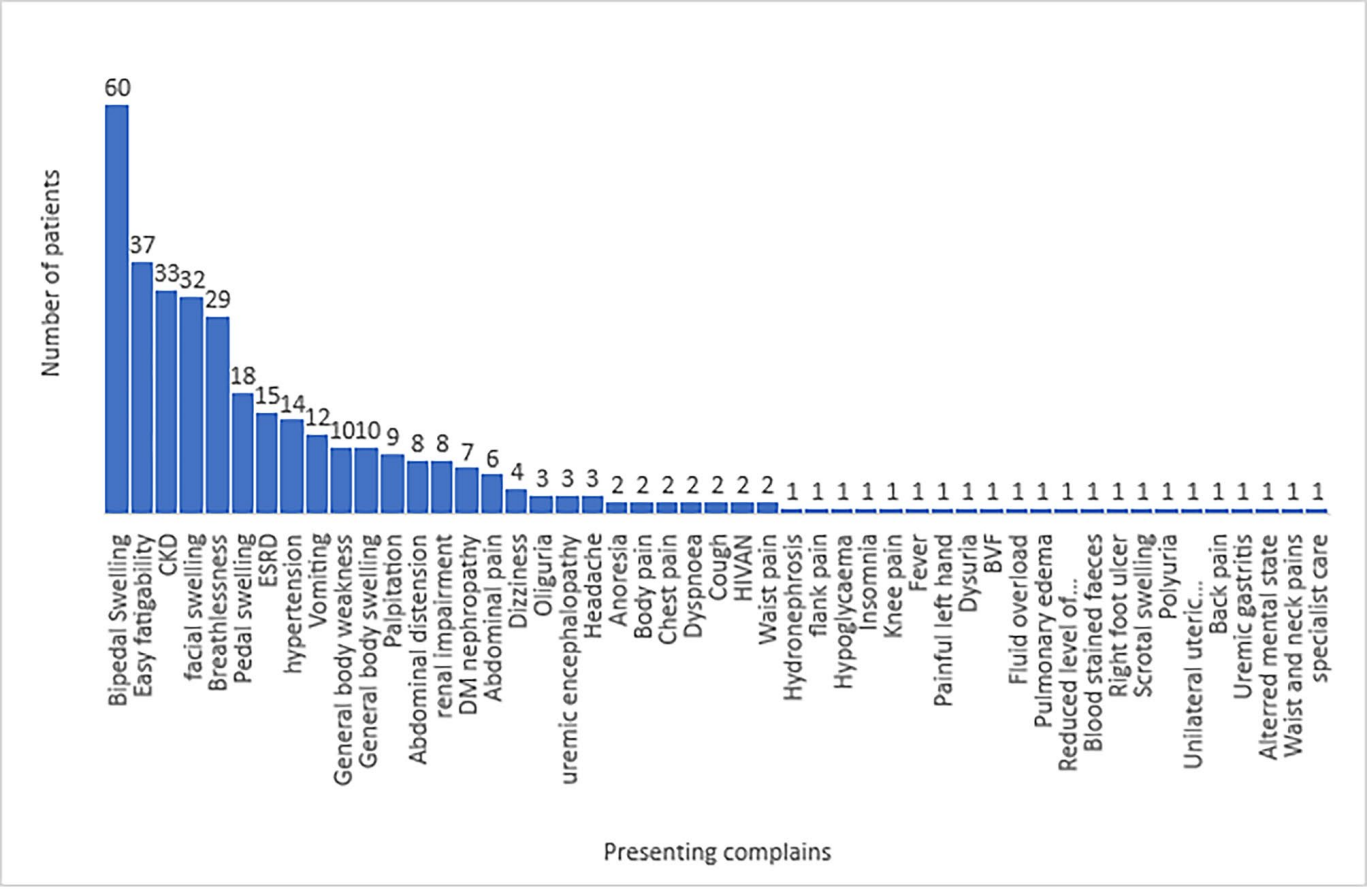
Presenting Complains of the participants ESRD: end stage renal disease, CKD: chronic kidney disease, HIV AN: HIV associated nephropathy, BVF: bilateral vestibular failure

The presenting complaints of the study patients are enumerated in Fig. 1. The major clinical features were bipedal swelling in 60/357 (16.8%), followed by easy fatigability in 37/357 (10.4%) and CKD in 33/357 (9.2%) patients. Thirty-two patients (9.2%), 29/357 (8.1%) and 18/ 357 (5.0%) presented with early morning facial swelling, breathlessness and pedal swellings respectively.

Hypertension (59.6% vs. 56.8%, p < 0.001) and hypertensive plus diabetes mellitus (32.7% vs. 28.0%, p < 0.001) were significant in RRT group contrary to the conservative group. The two groups also differed in their therapeutic characteristics as well as the comorbidities presented at diagnosis as summarized in Table 2.

**Table 2:** Cause of diagnosis, co-morbidity and therapeutic characteristics of the study patients

Variables	All patients	Renal replacement therapy group	Conservative group	P-value
	N (%)	N (%)	N (%)	
**Cause of diagnosis**				
CGN	119(31.4)	12(20.3)	107(33.4)	**< 0.001**
Hypertension	115(30.3)	19(32.2)	96(30.0)	**< 0.001**
Diabetes mellitus nephropathy	107(28.2)	24(40.7)	83(25.9)	**< 0.001**
HIVAN	12(3.2)	0(0.0)	12(3.8)	-
ADPKD	10(2.6)	3(5.1)	7(2.2)	0.206
Obstructive uropathy	8(2.1)	1(1.7)	7(2.2)	**0.034**
Others^a^	8(2.1)	0(0.0)	8(2.5)	-
**Co-morbid at the time of presentation**				
Hypertension	169(57.3)	31(59.6)	138(56.8)	**< 0.001**
Diabetes mellitus	20(6.8)	4(7.7)	16(6.6)	**0.007**
Hypertension with Diabetes Mellitus	85(28.8)	17(32.7)	68(28.0)	**< 0.001**
HIV	13(4.4)	0(0.0)	13(5.3)	-
Others^b^	8(2.7)	0(0.0)	8(3.3)	-
**Medications (yes)**				
Nifedipine	288(75.4)	50(82.0)	238(74.1)	**< 0.001**
Irbesartan/Lisinopril	218(57.1)	32(52.5)	186(57.9)	**< 0.001**
Bisoprolol	61(16.0)	20(32.8)	41(12.8)	**0.007**
Aspirin	41(10.7)	12(19.7)	29(9.0)	**0.008**
Prednisolone	14(3.7)	4(6.6)	10(3.1)	0.109
Ranitidine	70(18.3)	16(26.2)	54(16.8)	**< 0.001**
Metformin	34(8.9)	8(13.1)	26(8.1)	**0.002**
Gliclazide	382(100.0)	61(100.0)	321(100.0)	**< 0.001**
Lasix	295(77.2)	48(78.7)	247(76.9)	**< 0.001**
Hydralazine	139(36.4)	32(52.5)	107(33.3)	**< 0.001**
Methyldopa	191(50.0)	32(55.7)	157(48.9)	**< 0.001**
EPO	35(9.2)	17(27.9)	18(5.6)	0.866
CaCO_3_	242(63.4)	39(63.9)	203(63.2)	**< 0.001**
NaHCO_3_	193(50.5)	26(42.6)	167(52.0)	**< 0.001**
Statin	92(24.1)	19(31.1)	73(22.7)	**< 0.001**

**CGN: chronic glomerulonephritis, HIVAN: HIV associated nephropathy, HIV: human immunodeficiency virus, ADPKD: autosomal dominant polycystic kidney disease, EPO: erythropoietin, CaCO**_**3**_: **Calcium carbonate, NaHCO**_**3**_: **Sodium hydro carbonate, Others**^**a**^ : **Benign Prostatic Hyperplasia, Bilateral Vascular Failure, Diabetes Mellitus/Sickle Cell, Hepatitis B/HIV, Hypertension/Stroke, Sickle cell, Others**^**b**^ : **Chronic Interstitial nephritis, Chronic schistosomiasis, HIV, Multiple myeloma, Renal oncocytoma, Uremic gastritis, SC nephropathy**

Table 3 indicates that the median diastolic blood pressure and urea levels were significantly higher in the conservative treatment group compared with the RRT group. The chi square test also revealed that more RRT patients tested positive for protein in urine than the conservative treatment group (93.1% vs. 92.0%, p < 0.001). However, the conservative treatment group compared to the RRT group tested positive for blood in urine (56.4% vs. 50.0%, p < 0.001). There were no significant differences in SBP, hemoglobin, white blood cell count, MCV, MCH, sodium, potassium, triglycerides, LDL-c, HDL-c, VLDL-c, cholesterol, creatinine levels and eGFR between the two groups.

**Table 3:** Baseline Clinical and Laboratory investigation results of the patients before treatment

Parameters	All Subjects	Renal replacement therapy group	Conservative group	P value
	mean ± SD/ Median [IQR]	Mean ± SD/Median [IQR]	mean ± SD/Median [IQR]	
SBP (mmHg)	154.85 ± 25.1	152.6 ± 25.3	155.3 ± 25.1	0.459
DBP (mmHg)	90.0 [79.0-100.0]	83.3 [78.3–93.8]	90.0 [79.3-100.1]	**0.047**
Haemoglobin	8.0 [6.9–9.2]	8.3 [7.2–9.1]	7.9 [6.8–9.2]	0.309
White Blood Count	5.6 [4.6–7.1]	5.8 [5.0-7.2]	5.6 [4.5–7.1]	0.136
MCV	82.6 ± 6.8	83.11 ± 6.4	82.5 ± 6.9	0.557
MCH	27.67 ± 2.6	27.7 ± 2.6	27.7 ± 2.6	0.858
Sodium	135.7 [132.6–138.0]	136.0 [136.0-139.0]	135.5 [131.6–138.0]	0.159
Potassium	5.0 [4.5–5.6]	4.9 [4.0-5.4]	5.1 [4.5–5.6]	0.093
Urea	28.0 [20.0-37.4]	22.2 [15.0-30.3]	28.6 [20.9–38.0]	**0.001**
Triglycerides	1.3 [1.0-1.8]	1.2 [1.0-1.5]	1.3 [1.0–2.0]	0.566
LDL-c	3.1 [2.2–3.8]	2.9 [2.0-4.1]	3.1 [2.3–3.8]	0.809
HDL-c	1.2 [0.8–1.5]	1.2 [0.8–1.6]	1.1 [0.8–1.4]	0.945
VLDL-c	0.6 [0.4-1.0]	0.6 [0.4–0.8]	0.6 [0.5–1.1]	0.368
Cholesterol	5.0 [3.9–5.8]	5.0 [3.6–6.1]	5.0 [4.0-5.8]	0.593
Creatinine	1095.0 [703.4-1581.5]	1004.5 [570.8-1678.8]	1116.9 [714.0-1563.0]	0.333
eGFR	4.0 [3.0–7.0]	5.0 [3.0-8.3]	4.0 [3.0–7.0]	0.433
**Positive**	N (%)	N (%)	N (%)	
Urine Protein	187(92.1)	27(93.1)	160(92.0)	**< 0.001**
Urine Blood	105(55.6)	12(50.0)	93(56.4)	**< 0.001**

**SBP: systolic blood pressure, DBP: diastolic blood pressure, MCV: mean corpuscular volume, MCH: mean corpuscular hemoglobin, LDL-c: low density lipoprotein cholesterol, HDL-c: high density lipoprotein cholesterol, VLDL-c: very low-density lipoprotein cholesterol, eGFR: estimated glomerular filtration rate**

The complications after dialysis, conservative treatment and treatment outcomes are summarized in Table 4. Relative to the RRT group, more conservative group patients developed anemia (57.3% vs. 55.7%, p < 0.001), hyperkalemia (11.5% vs. 4.9%, p < 0.001) and uremic gastritis (3.1% vs. 1.6%, p = 0.007). However, the RRT patients had more fluid overload (18.0% vs. 12.8%, p < 0.001) and pulmonary edema (8.2% vs. 6.3% p < 0.001) compared with the conservative group.

**Table 4:** Complications after treatment and outcome in the study participants

	All patients	Renal replacement therapy group	Conservative group	p-value
	N (%)	N (%)	N (%)	
**Complications**				
Anaemia	218(57.1)	34(55.7)	184(57.3)	**< 0.001**
Fluid Overload	52(13.6)	11(18.0)	41(12.8)	**< 0.001**
Hyperkalaemia	40(10.5)	3(4.9)	37(11.5)	**< 0.001**
Pulmonary oedema	29(7.6)	5(8.2)	24(6.3)	**< 0.001**
Uncontrolled hypertension	24(6.3)	6(9.8)	18(5.6)	**0.014**
Uremic Gastritis	11(2.9)	1(1.6)	10(3.1)	**0.007**
Uremic encephalopathy	7(1.8)	1(1.6)	6(1.9)	0.059
Acute rejection	1(0.3)	0(0.0)	1(0.3)	-
**Outcome**				
Dead	8(2.1)	6(9.8)	2(0.6)	0.289
Alive	30(7.3)	12(19.7)	18(5.6)	0.273
Lost to follow-up	337(88.2)	41(67.2)	296(92.2)	**< 0.001**
Others	7(1.8)	2(3.3)	5(1.6)	**0.045**

In the bivariate logistic regression (first model), patients aged 40–54 years had lower odds of undergoing conservative treatment compared with those aged 10–24 years (OR: 0.242, 95% C.I. 0.037,0.731). Formal sector workers had less likelihood of opting for conservative treatment compared to unemployed patients (OR: 0.295, 95% C.I. 0.155, 0.754). The likelihood of undergoing conservative treatment was higher among patients diagnosed with ESKD within 1–5 months compared with those diagnosed in < 1 month (AOR: 3.854, 95% CI; 1.414, 10.506). Gender and occupation of the patients were not associated with the decision to opt for conservative treatment(p > 0.05). Controlling for confounding factors in the multivariate analysis, only duration of illness was significantly associated with the choice for conservative treatment option among the study participants. Thus, patients who were diagnosed within 1–5 months had higher likelihood of opting for conservative treatment compared with those diagnosed in < 1 month (AOR: 4.52, 95% CI; 1.350,15.162) (Table 5).

**Table 5:** Variables associated with the decision to undergo of conservative treatment

	Bivariate analysis		Multivariate analysis	
	OR (95% CI)	p-value	OR (95% CI)	p-value
**Age** [ref: 10–24 years]				
25–39	0.248(0.053,1.164)	0.077	0.257(0.014,4.620)	0.356
40–54	0.166(0.037,0.731)	0.018*	0.075(0.003,1.609)	0.098
55–69	0.242(0.054,1.087)	0.064	0.109(0.005,2.2860)	0.153
≥ 70	0.403(0.063,2.575)	0.337	_-	-
**Gender** [ref: Female]				
Male	0.623(0.347,1.119)	0.113	0.517(0.165,1.618)	0.257
**Religion** [ref: Muslim]				
Christian	1.412(0.585,3.405)	0.443	-	-
**Occupation** [ref: unemployed]				
students	1.987(0.508,7.779)	0.324	-	-
Pensioners	1.442(0.285,7.312)	0.658	-	-
Informal sector	1.228(0.556,2.714)	0.612	-	-
Formal sector	0.295(0.155,0.754)	0.011*	1.547(0.262,9.144)	0.630
**Duration of illness** [ref: <1 month]				
1–5	3.854(1.414,10.506)	0.008*	4.524(1.350,15.162)	0.014*
> 6	1.944(0.480,7.869)	0.351	-	-

## Discussion

This study compared the sociodemographic features, diagnostic characteristics (clinical, biochemical and imaging) and clinical outcomes of ESKD patients who chose either RRT or conservative therapy as well as the factors that influenced their choice. Bipedal swelling (16.8%), fatigue (10.4%) and facial swelling (9.2%) were the major clinical features. Chronic glomerulonephritis (31.4%), hypertension (30.3%) and diabetes mellitus nephropathy (28.2%) were the most frequent predisposing conditions. Nifedipine (82.0%), bisoprolol (32.8%), aspirin (19.7%), ranitidine (26.2%), metformin (13.1%) and lasix (78.7%) were commonly used by the RRT patients than their conservative therapy counterparts. Compared to their RRT counterparts, patients on conservative therapy were more on irbesartan/lisinopril (57.9%) and sodium hydro carbonate (NaHCO_3_) (52.0%). Diastolic blood pressure (DBP) (p = 0.047), uremic gastritis (p = 0.007), anaemia, uraemia, haematuria and hyperkalaemia (p < 0.001) were more common in conservative therapy patients than RRT patients with RRT patients showing better corticomedullary differentiation (38.1% vs. 27.7%, p < 0.001) and normal echotexture (15.0% vs. 11.6%, p = 0.005). In agreement with previous studies, majority of our participants were aged 40–69 years with a male preponderance [20, 21]. ESKD is known to occur in advanced ages where risk factors are high with more comorbidities [22]. The male preponderance is likely due to hypertension, a major risk factor, occurring more in males than in females with the incidence of uncontrolled BP often in males[23]. Male prevalence in incidents of primary renal diseases [11, 24] also plays a role.

At diagnosis, the most prevalent causes of ESKD were CGN, hypertension and diabetes nephropathy (Table 2) with majority of the participants presenting with pedal, facial and bodily swelling as well as fatigue (Fig. 1) as observed in other studies [25][20]. Conservative therapy patients had higher occurrence of uremia, hyperkalemia and hematuria with more DBP measurements. A lot of these patients were on ACEis/ARBs which are known to reduce GFR and cause some electrolyte imbalances as their adverse effects [26] could explain the uremia and hyperkalemia observed.

Complications like uncontrolled hypertension, anemia (Table 4) and hematuria (Table 3) are all associated with ESKD progression [5][27][10]. Hence, from our data, we can infer that disease progression is faster in the conservative therapy participants than the RRT participants. Ergo, the RRT patients showed better corticomedullary differentiation and normal echotexture (Table 5). Nevertheless, some complications such as fluid overload and pulmonary edema slightly dominated in the RRT patients. These are common occurrences in dialysis patients [28, 29] mostly resulting from inaccuracies in the dialysis process and patient lifestyle [30].

One major objective in CKD and ESKD management is to control BP to a target level of < 130/80 mmHg [27], which is why blood pressure drugs dominated among the medication administered. Nifedipine (a calcium channel blocker) was the most used BP drug among hydralazine, methyldopa and bisoprolol in both groups because they are highly bound and excreted through hepatic metabolism hence unaffected by kidney dysfunction. Other drugs administered include gliclazide and metformin for Type 2 diabetes mellitus, CaCO_3_, fersolate and folic acid as nutrient supplements, aspirin and prednisolone for pain and allergies among others.

Ideally, ESKD is managed with dialysis or kidney transplantation and sometimes conservative therapy for very old patients with many comorbidities [31]. We noted that none of our participants had kidney transplant. Also, majority of our patients were on conservative therapy rather than RRT (Table 1). This finding proves the point made by Antwi, (2015) [2] that “the current state of RRT services in Ghana is inadequate and calls for serious national consideration”. In his paper, he attributed this to several reasons including limited dialysis facilities in the country, unavailability of insurance schemes to cover cost despite the high cost of these therapies and lack of constant electricity supply. We conducted this study in one of the only 6 public dialysis centers available in the country, all of which are in the southern zone. This shows how scarce the treatment is to the general public especially those in the northern and central zones. Most people who opt for RRT may have to travel several kilometers away from home to access treatment. Considering the fact that majority of our participants are either unemployed or in the informal sector (Table 1) and with the unavailability of insurance coverage for citizens, the collective cost of travelling, laboratory investigations, drugs as well as dialysis sessions is unaffordable to most of them and they would have to settle for conservative therapy.

From Table 4, we noticed that age and gender are variables significantly associated with the decision to opt for conservative therapy. Looking at the majority age range (40–69 years) with most being males, it is likely that these individuals are breadwinners with many financial responsibilities and may not have the luxury or support to finance RRT. Duration of illness was also a significant variable; more of the patients who had had the disease for longer periods opted for conservative therapy. This is likely because hemodialysis is an awfully burdensome intervention especially in Ghana and chronic hemodialysis patients often opt out and resort to conservative care as disease progresses and resources are depleted.

A major limitation in our study was that some participants did not complete their data and were disqualified. Thus, we lost about 80% of our participants to follow up and we could also not access and contrast the quality of life of both groups during treatment.

## Conclusion

In this study, ESKD progression occurred faster in patients who opted for conservative therapy because it was associated with worse clinical features and biochemical derangements than Renal Replacement Therapy. The major factors that influenced the choice of either RRT or conservative care were age, gender and duration of illness. Duration of illness was the only significant factor after controlling for the other variables. Hence early referrals to the nephrologist should be encouraged. Also, policy makers should aim at increasing dialysis centers in the country while providing substantial financial support to chronic dialysis patients.

## Data Availability

The Datasets used for this study are not publicly available because they contain information that could compromise the privacy of the study participants but are available from the corresponding author on reasonable request.

## List of abbreviations

CKD: chronic kidney disease.
ESRD: end stage kidney disease.
RRT: renal replacement therapy.
KATH: Komfo Anokye Teaching Hospital.
CGN: chronic glomerulonephritis.
HIVAN: HIV associated nephropathy.
HIV: human immunodeficiency virus.
ADPKD: autosomal dominant polycystic kidney disease.
EPO: erythropoietin.
CaCO_3_: Calcium carbonate.
NaHCO_3_: Sodium hydro carbonate. SBP:systolic blood pressure.
DBP: diastolic blood pressure.
MCV: mean corpuscular volume.
MCH: mean corpuscular hemoglobin.
LDL-c: low density lipoprotein cholesterol.
HDL-c: high density lipoprotein cholesterol.
VLDL-c: very low-density lipoprotein cholesterol.
eGFR: estimated glomerular filtration rate.
LPP: pole-to-pole kidney length.
PW: parenchymal width.
